# New closed-loop insulin systems

**DOI:** 10.1007/s00125-021-05391-w

**Published:** 2021-02-06

**Authors:** Charlotte K. Boughton, Roman Hovorka

**Affiliations:** Wellcome Trust-Medical Research Council Institute of Metabolic Science, University of Cambridge, Cambridge, UK

**Keywords:** Artificial pancreas, Automated insulin delivery, Hybrid closed-loop, Review, Type 1 diabetes

## Abstract

Advances in diabetes technologies have enabled the development of automated closed-loop insulin delivery systems. Several hybrid closed-loop systems have been commercialised, reflecting rapid transition of this evolving technology from research into clinical practice, where it is gradually transforming the management of type 1 diabetes in children and adults. In this review we consider the supporting evidence in terms of glucose control and quality of life for presently available closed-loop systems and those in development, including dual-hormone closed-loop systems. We also comment on alternative ‘do-it-yourself’ closed-loop systems. We remark on issues associated with clinical adoption of these approaches, including training provision, and consider limitations of presently available closed-loop systems and areas for future enhancements to further improve outcomes and reduce the burden of diabetes management.

**Supplementary Information:**

The online version contains a slideset of the figures for download available at 10.1007/s00125-021-05391-w.

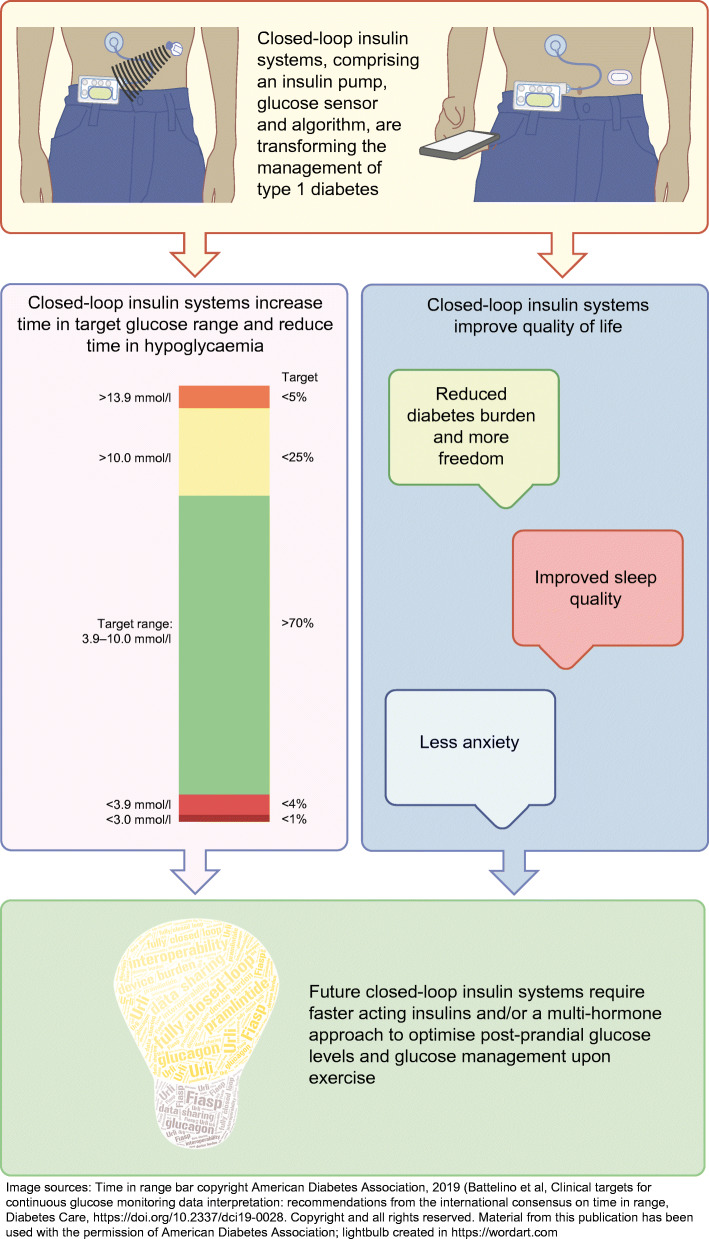

## Introduction and development of closed-loop systems

**Fig. 1:**
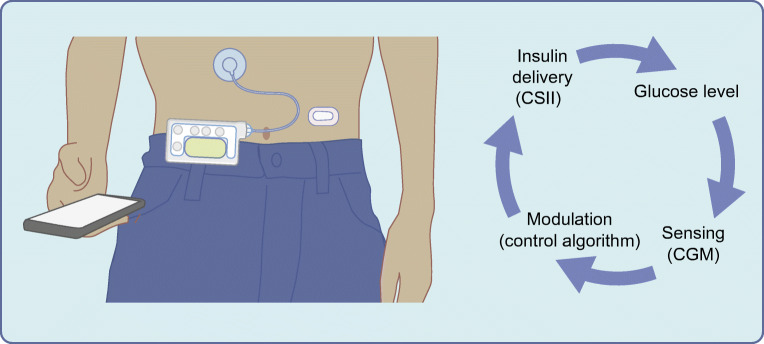
Schematic of the configuration of closed-loop insulin delivery. A CGM transmits information about interstitial glucose concentrations to an algorithm hosted on a smartphone or insulin pump that translates information from the glucose sensor and computes the amount of insulin to deliver. An insulin pump delivers a rapid-acting insulin analogue subcutaneously. Insulin delivery is modulated in real time by the control algorithm. Communication between system components is wireless. CSII, continuous subcutaneous insulin infusion. Figure adapted from [36]. This figure is available as part of a downloadable slideset

Hybrid closed-loop insulin delivery systems are gradually transforming clinical management of type 1 diabetes. They comprise a subcutaneously worn continuous glucose monitor (CGM or glucose sensor) device, communicating with an algorithm that responds in real time to changes in sensor glucose levels, and adjusts the subcutaneous insulin infusion delivered by an insulin pump (Fig. 1).

**Fig. 2:**
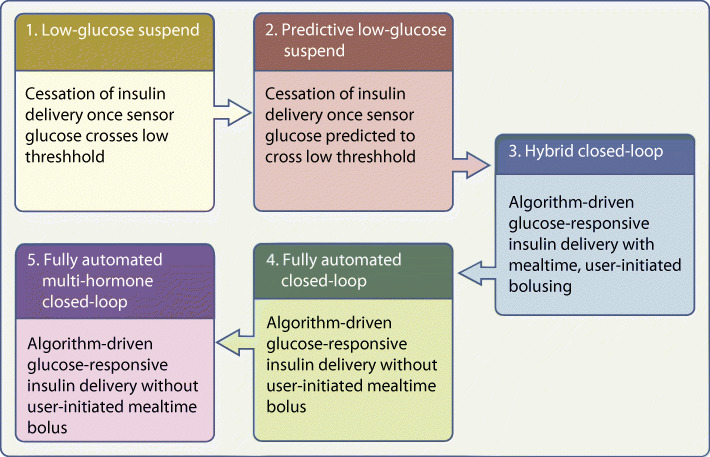
Key developmental milestones towards a truly artificial pancreas. This figure is available as part of a downloadable slideset

Although the concept of glucose-responsive insulin delivery has been around for 50 years, earlier developments of closed-loop systems were hampered by the lack of accurate and reliable CGM systems, unavailability of wearable computational devices and secure wireless communication protocols, and challenged by limitations in insulin pump devices. With advances in CGM technology, the simplest form of automated insulin delivery was achieved with low-glucose suspend systems, where insulin delivery is suspended when the sensor glucose crosses a specified threshold, and predictive glucose management systems, where insulin delivery is suspended when an algorithm predict*s* that sensor glucose is likely to cross the low-glucose threshold [1, 2]. These systems reduce hypoglycaemia, although sometimes at the expense of increased hyperglycaemia [3]. These were conceptually landmark steps in the journey towards fully automated insulin delivery and a true ‘artificial pancreas’ (Fig. 2).

**Fig. 3:**
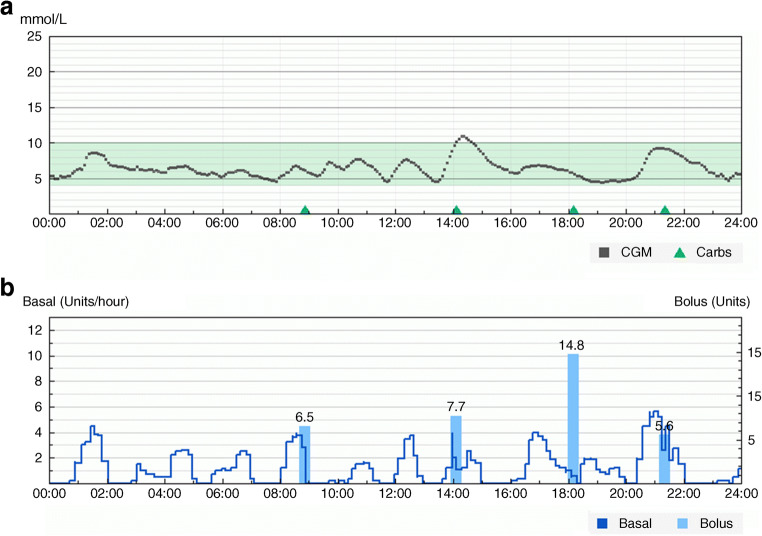
Hybrid closed-loop glucose control. (**a**) 24 h of sensor glucose data. Green shaded area is the target glucose range (3.9–10 mmol/l). Green triangles indicate carbohydrate (carbs) intake. (**b**) Algorithm-driven insulin delivery and manual insulin boluses. Data in both graphs derived from a Cambridge closed-loop study participant (the individual provided permission to share this anonymised data for the purpose of advancement of science). The graphs were generated using the data management platform Diasend (https://diasend.com//en). Image credit: Glooko Inc. All rights reserved. 2021. The *x*-axes show time in hours. This figure is available as part of a downloadable slideset

A closed-loop system is a more sophisticated system, with a control algorithm adjusting insulin delivery (up and down) in response to real-time sensor glucose levels and other inputs, such as meal intake (Fig. 3). The algorithm can accommodate variability of insulin requirements between and within individual users, and account for limitations of CGM accuracy and imprecisions of subcutaneous insulin delivery. Adaptation of the control algorithm to changes in physiological conditions with real-time adjustment of closed-loop control parameters is beneficial for optimal performance. Several different types of control algorithm have been developed, including model predictive control (MPC) algorithms, proportional integral derivative (PID) controllers and fuzzy logic control approaches [4]. MPC algorithms calculate insulin delivery by minimising the difference between model-predicted glucose concentrations and target glucose over a pre-specified prediction time horizon. PID controllers adjust insulin delivery by assessing glucose excursions from three perspectives: (1) deviation from target glucose (proportional component); (2) area under the curve between measured and target glucose (integral component); and (3) rate of change of measured glucose (derivative component). The fuzzy logic approach modulates insulin delivery based on approximate rules to express empirical knowledge of diabetes practitioners.

The first commercial closed-loop system, the MiniMed 670G (Medtronic, Northridge, CA, USA) was approved by the US Food and Drug Administration in September 2016 for use in people with type 1 diabetes aged 14 years and older [5]. This hybrid closed-loop system requires users to manually enter prandial insulin with automation of insulin delivery between meals and overnight. Several other hybrid closed-loop systems have since been commercialised and are increasingly being utilised in routine clinical care for people with type 1 diabetes.

## Efficacy and safety of hybrid closed-loop systems

Clinical studies evaluating the safety and efficacy of hybrid closed-loop systems have evolved from small, highly supervised studies, undertaken overnight or over 24 h in research facilities, to larger randomised controlled trials of unrestricted home living use, conducted over 6 months or longer. A meta-analysis, published in 2018, of 40 early outpatient studies, reports the efficacy and safety of hybrid closed-loop systems in people with type 1 diabetes [6]. Earlier closed-loop systems were associated with a 9.6 percentage point improvement in time in target glucose range (3.9–10.0 mmol/l) compared with comparator therapies (>2 additional h/day) and reduced time in hypoglycaemia (<3.9 mmol/l) by 1.5 percentage points (approximately 20 min/day) compared with control treatment. Hybrid closed-loop systems have a favourable effect on HbA_1c_, with a reduction of 0.3–0.4% compared with control therapy in studies with a duration of more than 8 weeks per intervention. While this effect appears modest, this is despite the reduction in hypoglycaemia observed in several of these studies, as well as the low HbA_1c_ at recruitment, reflecting good baseline glycaemic control of study participants. Similar benefits have been reported in a meta-analysis of 25 studies in the paediatric population [7].

Individual randomised controlled trials demonstrate glycaemic benefits of hybrid closed-loop systems vs comparator therapies, but comparisons of efficacy between hybrid closed-loop systems across different studies is hampered by variation in baseline characteristics of participants, study duration and design. Studies including participants with variable experience in diabetes technology use (on multiple daily insulin injections and without previous sensor use) and from more diverse socioeconomic backgrounds are important to support generalisability of benefits.

## Psychosocial impact of closed-loop systems

The impact of closed-loop technology on quality-of-life measures has been explored in several studies [8]. Psychosocial benefits reported by users include reduced anxiety, improved sleep and confidence from improved overnight glucose control, less restrictive eating habits and ‘time off’ from the demands of diabetes management. Reported challenges include technical issues, alarm intrusiveness and equipment burden, in addition to initial difficulties trusting the system. Most participants in closed-loop studies report that they would continue using closed-loop therapy or would recommend it to others as the clinical benefits outweigh system shortcomings. Psychosocial studies have largely included participants involved in closed-loop trials who may not be representative of the wider population living with type 1 diabetes.

## Commercially available closed-loop systems

Details of the commercially available hybrid closed-loop systems can be found in Table 1 and an overview of key clinical studies are shown in Table 2.

**Table 1:** Commercially available hybrid closed-loop systems

Component	Medtronic 670G	Medtronic 780G	CamAPS FX	Control-IQ
Algorithm	PID with insulin feedback	PID with insulin feedback	Treat to target adaptive MPC (Cambridge algorithm)	Treat to range predictive algorithm
Insulin pump	670G	780G	Dana RS, Dana-i	Tandem t:slim X2
CGM system	Guardian 3 (requires ~4–6 fingersticks/day)	Guardian 3 (requires ~4 fingersticks/day)	Dexcom G6 (factory calibrated, optional calibration)	Dexcom G6 (factory calibrated, optional calibration)
Target glucose	Fixed target: 6.7 mmol/l	Target: 5.6 mmol/l (default) or 6.7 mmol/l	Target: 5.8 mmol/l (default); customisable between 4.4 mmol/l and 11 mmol/l	Fixed target range: 6.2–8.9 mmol/l
	Optional activity target	Optional activity target		Night mode: 6.2–6.7 mmol/l
			Optional activity target	Optional activity target
Algorithm learning	Based on TDD	Based on TDD	Adapts to prandial and diurnal patterns	None
Compatible downloading software	Carelink; manual downloading of pump required	Carelink; automated app compatibility	Diasend; automated download	Clarity: sensor data
				Diasend/Glooko; manual downloading of pump required

TDD, total daily dose

**Table 2:** Key clinical studies for commercially available hybrid closed-loop systems

Closed-loop device [study reference]	Study design	Study duration	Population	Baseline HbA_1c_	Glucose outcomes
Medtronic 670G [9]	Non-randomised before-and-after single-arm study	3 months	*n* = 30 adolescents, ≥14 years old; *n* = 94 adults	Adolescents: 7.7% (61 mmol/mol); adults: 7.3% (56 mmol/mol)	Adolescents:
					• TIR ↑ from 60% (baseline) to 67%
					• TBR ↓ from 4.3% (baseline) to 2.8%
					Adults:
					• TIR ↑ from 69% (baseline) to 74%
					• TBR ↓ from 6.4% (baseline) to 3.4%
Medtronic 670G [10]	Non-randomised before-and-after single-arm study	3 months	*n* = 105 children (7–13 years old)	7.9% (63 mmol/mol)	• TIR ↑ from 56% (baseline) to 65%
					• TBR ↓ from 4.7% (baseline) to 3.0%
Medtronic 780G (AHCL) [11]	Randomised crossover study comparing Medtronic AHCL with 670G	3 months	*n* = 113 adolescents and young adults (14–29 years old)	7.9% (63 mmol/mol)	AHCL vs 670G:
					• TIR ↑: 67% vs 63%
					• TBR ↔: 2.1% vs 2.1%
Control-IQ [12]	Randomised parallel study comparing Control-IQ with SAP	6 months	*n* = 168 adults and adolescents ≥14 years old	7.4% (57 mmol/mol)	Control-IQ vs SAP:
					• TIR ↑: 71% vs 59%
					• TBR ↓: 1.6% vs 2.3%
Control-IQ [13]	Randomised parallel study comparing Control-IQ with SAP	4 months	*n* = 101 children (6–13 years old)	7.6–7.9% (60–63 mmol/mol)	Control-IQ vs SAP:
					• TIR ↑: 67% vs 55%
					• TBR ↔: 1.6% vs 1.8%
Cambridge closed-loop [16]	Randomised parallel study comparing closed loop with SAP	3 months	*n* = 86 children and adults with sub-optimal glycaemic control	7.8–8.0% (62–64 mmol/mol)	Closed loop vs SAP:
					• TIR ↑: 65% vs 54%
					• TBR ↓: 2.6% vs 3.9%
Cambridge closed-loop [17]	Randomised crossover study comparing closed-loop using diluted insulin with closed-loop using standard-strength insulin	3 weeks	*n* = 24 children (2–7 years old)	7.4% (57 mmol/mol)	For both groups:
					• TIR: 70–72%
					• TBR: 4.5–4.7%

SAP, sensor-augmented pump; TBR, time below range (<3.9 mmol/l); TIR, time in range (3.9–10 mmol/l)

### Medtronic 670G and 780G

Safety and efficacy of the first commercially approved hybrid closed-loop system (Medtronic 670G insulin pump with Guardian 3 sensor) was evaluated in a non-randomised before-and-after study in 30 adolescents and 94 adults with type 1 diabetes, over 3 months (Table 2) [9]. The proportion of time spent in target glucose range (3.9–10 mmol/l) increased from 60% at baseline to 67% with the closed-loop system in adolescents, and from 69% to 74% in adults. Time spent in hypoglycaemia (<3.9 mmol/l) was reduced with the closed-loop system and there were no episodes of severe hypoglycaemia or diabetic ketoacidosis in the study. A similar before-and-after study evaluating use of this device in 105 younger children aged 7–13 years reported increased time in target range and reduced time in hypoglycaemia with the closed-loop system (Table 2) [10].

A second-generation advanced hybrid closed-loop (AHCL [780G, Medtronic]) system has been developed to further improve glycaemic control and usability, with adjustable target glucose and automated correction boluses. The Fuzzy Logic Automated Insulin Regulation (FLAIR) study directly compared the Medtronic 670G with the AHCL system in adolescents and young adults with type 1 diabetes. Time in target glucose range was higher with the AHCL system than with the Medtronic 670G, while time in hypoglycaemia was similar (Table 2). The AHCL system was associated with fewer system alerts, reduced Auto Mode exits and increased time spent in Auto Mode (86% vs 75%) [11].

### Tandem control-IQ

**Fig. 4:**
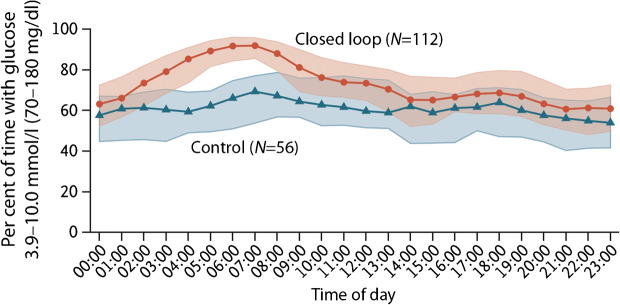
Median percentage time with sensor glucose in target range during closed-loop insulin delivery (red line) and sensor-augmented pump therapy (blue line) in adults and adolescents ≥14 years of age, using the Tandem Control-IQ closed-loop system [12]. The red and blue shaded areas indicate the interquartile range for each treatment. Figure adapted from [12]. Copyright: ©2019 Massachusetts Medical Society. Reprinted with permission from Massachusetts Medical Society. This figure is available as part of a downloadable slideset

In the longest randomised controlled closed-loop study to date, involving 168 people with type 1 diabetes (age ≥ 14 years), the Control-IQ system (t:slim X2 pump with Dexcom G6 sensor [Tandem, San Diego, CA, USA]) was compared with sensor-augmented pump therapy over 6 months (Table 2; Fig. 4) [12]. Time in target glucose range increased by 10 percentage points from baseline with the closed-loop system (61% to 71%) while there was no change in the control group (59% to 59%). Time spent in hypoglycaemia was reduced with the closed-loop system compared with the control group and there was an improvement in HbA_1c_. Notably, all participants completed the study, suggesting high acceptability of the technology.

In another study, the Control-IQ system was compared with sensor-augmented pump therapy in 101 younger children with type 1 diabetes, aged 6–13 years, over 16 weeks (Table 2). The closed-loop group had significantly greater improvements in time in target range, while the time spent in hypoglycaemia was similar between the closed-loop and control groups [13].

### CamAPS FX

CamAPS FX is the first interoperable hybrid closed-loop mobile phone application (app), utilising a control algorithm developed at the University of Cambridge (CamDiab; Cambridge, UK). At present, the app, hosted on an unlocked android smartphone, communicates with the Dana RS and Dana-i pumps and the Dexcom G6 sensor, but will communicate with additional pump and CGM systems in the future. The algorithm has been extensively evaluated in randomised controlled studies including children, adolescents, adults and pregnant women with type 1 diabetes [14, 15]. In a randomised controlled trial including 86 children and adults with sub-optimal glycaemic control (baseline HbA_1c_ > 58 mmol/mol [7.5%]), closed-loop use increased time in target range over 12 weeks compared with sensor-augmented pump therapy (Table 2) [16]. Time spent in hypoglycaemia was lower in the closed-loop group than the control group and HbA_1c_ also improved with closed-loop use.

Closed-loop insulin delivery using the Cambridge algorithm has been demonstrated to be feasible even in the very youngest of children, aged 1–7 years. In a study comparing the Cambridge closed-loop system using diluted insulin with the same closed-loop system using standard-strength insulin in children aged 2–7 years old, time in target glucose range was >70% and time in hypoglycaemia was <5% during both interventions. There were no safety or efficacy benefits of diluted insulin use compared with use of standard-strength insulin (Table 2) [17].

In a randomised controlled study involving 16 pregnant women with type 1 diabetes, closed-loop use overnight was associated with increased time in the tighter glucose range recommended during pregnancy (3.5–7.8 mmol/l) compared with sensor-augmented pump therapy (75% vs 60%), without increasing the risk of hypoglycaemia [14]. During the continuation phase, the closed-loop system was used 24 h/day, including during labour and delivery, and time in target glucose range was 69%.

## Anticipated commercial hybrid closed-loop systems

The Diabeloop hybrid closed-loop system, comprising a handset containing the algorithm, Kaleido pump and Dexcom G6 sensor (DBLG1; Diabeloop, Grenoble, France), has been compared with sensor-augmented pump therapy in 68 adults with type 1 diabetes, in the home setting, with remote monitoring over 12 weeks [18]. Time with glucose in target range was 9 percentage points greater with closed-loop therapy than with control therapy (69% vs 59%) and time in hypoglycaemia was significantly lower with closed-loop use than during the control period. The DBLG1 hybrid closed-loop system has received the CE mark for use in adults with type 1 diabetes and is due to be commercialised.

The Insulet Omnipod Horizon hybrid closed-loop system, comprising the Omnipod pump and Dexcom G6 sensor (Insulet, Billerica, MA, USA), and Beta Bionics insulin-only iLet hybrid closed-loop system, comprising the iLet Bionic Pancreas System and Dexcom G6 sensor (Beta Bionics, Boston, MA, USA), are both currently being evaluated in pivotal trials with launches anticipated in the next 1–2 years.

## Do-it-yourself closed-loop systems

The do-it-yourself (DIY) artificial pancreas system (DIYAPS) communities arose out of their frustration with the slow progress of medical device development cycles (the #wearenotwaiting movement). The communities develop and apply open-access closed-loop systems (e.g. the Open Artificial Pancreas System [OpenAPS], Loop and AndroidAPS), which do not undergo regulatory overview and approval. Without needing to await regulatory approval for new developments, these systems benefit from more rapid innovation cycles and can be more flexible in terms of customisation. In principle, access is open to anyone but users must be able to build and maintain their own system, albeit with support from the community itself. The role of healthcare providers in supporting the use of unregulated systems continues to be debated.

Several thousands of individuals use DIY systems globally. Observational before-and-after studies show improvements in time in target glucose range, HbA_1c_ and quality of life, but no longitudinal randomised controlled trials have evaluated the efficacy and safety of these systems [19]. A randomised clinical trial of a version of AndroidAPS is underway (ACTRN12620000034932p).

## Dual-hormone closed-loop systems

The physiological glucagon response to hypoglycaemia is often impaired in type 1 diabetes; therefore, addition of glucagon to a closed-loop system confers additional protection from hypoglycaemia and may allow more aggressive insulin delivery to achieve improved glucose control. Potential benefits are countered by increased system complexity, requirement for two separate infusion systems and the lack of approved room-temperature-stable glucagon for chronic subcutaneous delivery. There are currently no commercially available dual-hormone closed-loop systems, although several are in development.

The longest dual-hormone closed-loop home study, with remote monitoring, included 43 adults with type 1 diabetes with optional meal announcements over a period of 11 days. Time in target glucose range was increased (78% vs 62%) and hypoglycaemia (<3.3 mmol/l) was reduced (0.6% vs 1.9%) with dual-hormone closed-loop use compared with insulin pump therapy alone [20]. A shorter study, over 5 days, involving 32 adolescents with type 1 diabetes, demonstrated a 21 percentage point increase in time in target glucose range during the closed-loop period compared with the control period, but time in hypoglycaemia was similar between groups [21]. An outpatient study of over 60 h compared dual-hormone with single-hormone closed-loop systems in 23 adults with type 1 diabetes and showed no difference in time in target glucose range (79% vs 75%) or time in hypoglycaemia (<4.0 mmol/l; 3.6% vs 3.9%), but longer studies are required to fully investigate potential differences [22].

Pramlintide is an analogue of amylin, which is co-secreted with insulin from beta cells and reduces postprandial glucose excursions by slowing gastric emptying [23]. A novel dual-hormone closed-loop system delivering a fixed ratio of pramlintide:insulin was evaluated during a 24 h inpatient study in adults with type 1 diabetes. The dual-hormone system improved time in target range compared with an insulin-alone system (84% vs 74%), an effect attributable to improved daytime glucose control [24]. Gastrointestinal symptoms were reported more frequently during use of closed-loop systems with pramlintide as compared with insulin only. Pramlintide co-delivery may support the development of fully closed-loop systems, obviating the need for manually initiated prandial insulin delivery.

## Training considerations

High quality user and healthcare professional training is essential for ensuring that the clinical benefits of hybrid closed-loop systems are realised in the real-world setting. This is an important consideration for health economic analyses, to support adoption, implementation and reimbursement. Establishing realistic expectations of hybrid closed-loop therapy and reiterating the importance of core diabetes skills and tasks is important to promote long-term use and optimal clinical outcomes. Training programmes have been developed, using online and face-to-face approaches, to support users to maximise glycaemic and quality-of-life benefits of closed-loop therapy [25].

## Limitations of closed-loop systems

Early real-world use of the first commercially approved hybrid closed-loop system (Medtronic 670G) exposed issues around usability. The system required significant user input to remain in Auto Mode and, in one prospective observational study, one-third of users discontinued use of Auto Mode during the first year after initiation [26]. Factors influencing discontinuation include CGM issues (calibrations), number of alarms and efforts to limit Auto Mode exits [27]. Usability issues can prevent realisation of the benefits of closed-loop systems as increased time in Auto Mode is associated with improved glycaemic outcomes [28].

Some first-generation hybrid closed-loop systems use a relatively high glucose target (6.7 mmol/l) and lack flexibility to adjust the target to suit the needs of the user. This makes the system unsuitable for those aiming for tighter glycaemic control, including pregnant women.

Postprandial glucose excursions remain a challenge for closed-loop systems due to inherent delays in subcutaneous insulin absorption. User interaction with accurate carbohydrate counting and pre-meal bolusing is required for optimal glycaemic control. Attempts to reduce user burden with simplified meal boluses or fully closed-loop systems have resulted in compromised glycaemic control [29, 30].

Managing physical activity can be challenging primarily due to increased hypoglycaemia risk and altered insulin sensitivity. Even with closed-loop glucose-responsive insulin delivery, users usually need to plan for exercise, announcing exercise to the algorithm in advance, and may still require carbohydrate intake to prevent hypoglycaemia [31, 32]. Carbohydrate loading before exercise can be problematic with glucose-responsive insulin delivery, often resulting in hypoglycaemia during exercise.

Important ethical considerations include ensuring equitable access, training and support for closed-loop technology, and protecting user confidentiality and safety from security breaches [33].

## Future developments in automated insulin delivery

Future closed-loop systems will benefit from improved individual components; smaller, more accurate CGM devices with longer wear-time and smaller insulin pumps with the user interface transferred to a smartphone/watch will improve usability and minimise device burden. Interoperable devices and data management platforms offer flexibility for users to create their own personalised closed-loop ecosystem.

The introduction of new faster-acting insulin analogues (Fiasp and Ultra-Rapid Lispro) provides an opportunity to potentially improve performance of closed-loop systems with faster onset and offset of insulin action following subcutaneous delivery. Short studies comparing faster-acting insulin with standard insulin using the Medtronic 670G system have not shown significant benefits, but longer studies are required to fully evaluate closed-loop systems with faster-acting insulin [34, 35]. Faster-acting insulins are not yet approved for use in the t:slim X2 pump and, hence, the Control-IQ closed-loop system.

Integration of additional signals to algorithms, such as heart rate or accelerometery, to more quickly detect physical activity than with CGM alone, may reduce exercise-related hypoglycaemia. If efficacious, this would be particularly beneficial in young children in whom activity is usually spontaneous and unpredictable and hypoglycaemia is a major concern.

## Conclusions

The last 5 years has seen the successful transition of closed-loop systems from research into routine clinical practice for management of type 1 diabetes. There is still scope for further improvements to optimise postprandial glucose control, exercise management and usability before this technology can be said to truly ameliorate the burden of diabetes. Widespread adoption and reimbursement of closed-loop systems will be critical to ensuring equitable access to this technology.

## Supplementary Information

Slideset of figures (PPTX 768 kb)

## Abbreviations

AHCL: Advanced hybrid closed-loop
App: Application (mobile phone)
CGM: Continuous glucose monitor
DIY: Do-it-yourself
MPC: Model predictive control
PID: Proportional integral derivative
