# Correction to: New closed-loop insulin systems

**DOI:** 10.1007/s00125-021-05443-1

**Published:** 2021-03-22

**Authors:** Charlotte K. Boughton, Roman Hovorka

**Affiliations:** Wellcome Trust-Medical Research Council Institute of Metabolic Science, University of Cambridge, Cambridge, UK

**Correction to: Diabetologia**

10.1007/s00125-021-05391-w

This paper was published with Open Access. The copyright line has been updated to indicate that the authors retain copyright.

