# Supplementary material for: New closed-loop insulin systems
Source: Diabetologia. 2021 Feb 6;64(5):1007–15. doi: 10.1007/s00125-021-05391-w (PMC8012332; doi:10.1007/s00125-021-05391-w)
Supplement: Supplementary file 1 — (PPTX 768 kb) [file 125_2021_5391_MOESM1_ESM.pptx]

## Slide 1
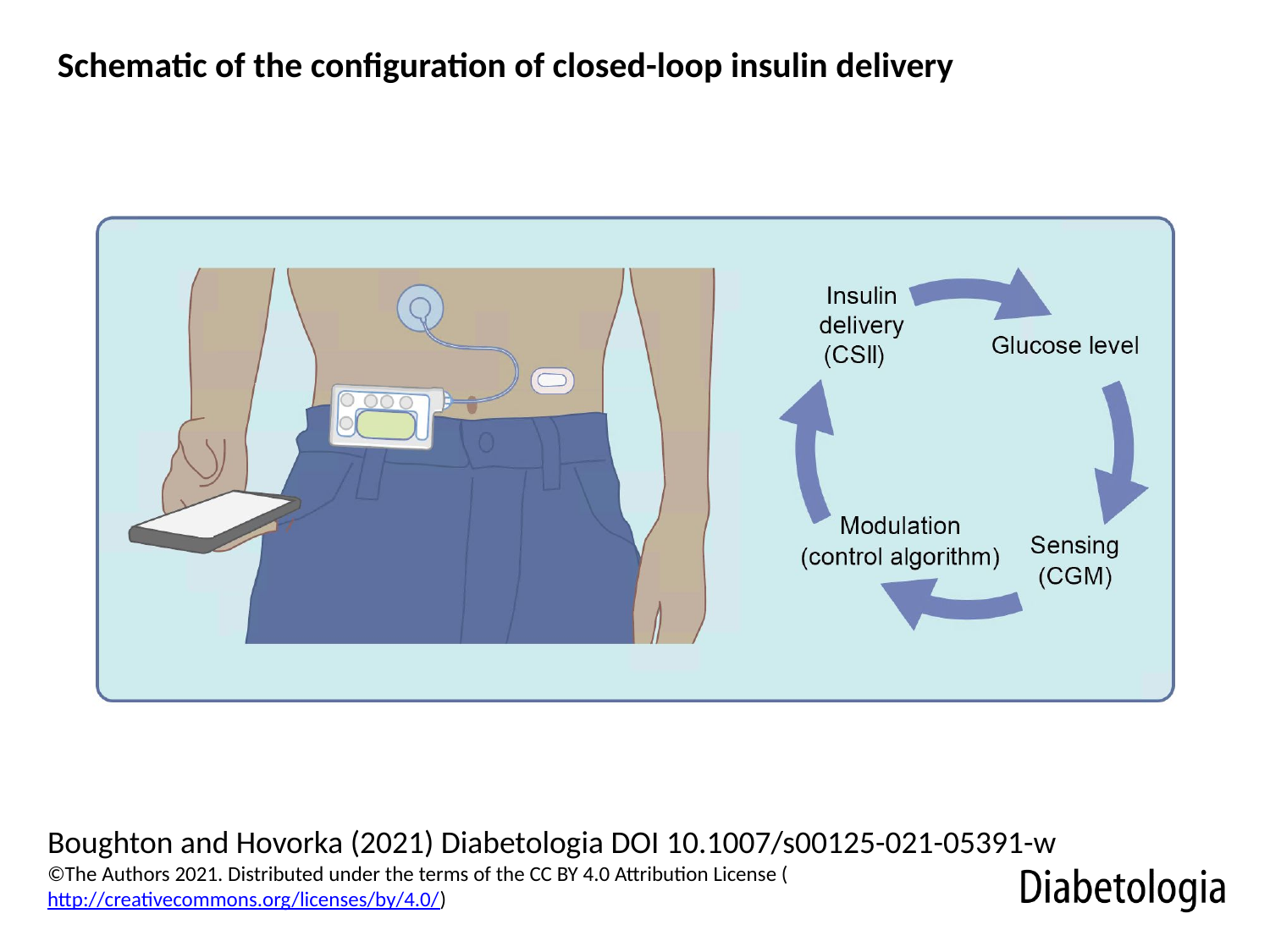

Schematic of the configuration of closed-loop insulin delivery
Boughton and Hovorka (2021) Diabetologia DOI 10.1007/s00125-021-05391-w
©The Authors 2021. Distributed under the terms of the CC BY 4.0 Attribution License (http://creativecommons.org/licenses/by/4.0/)

## Slide 2
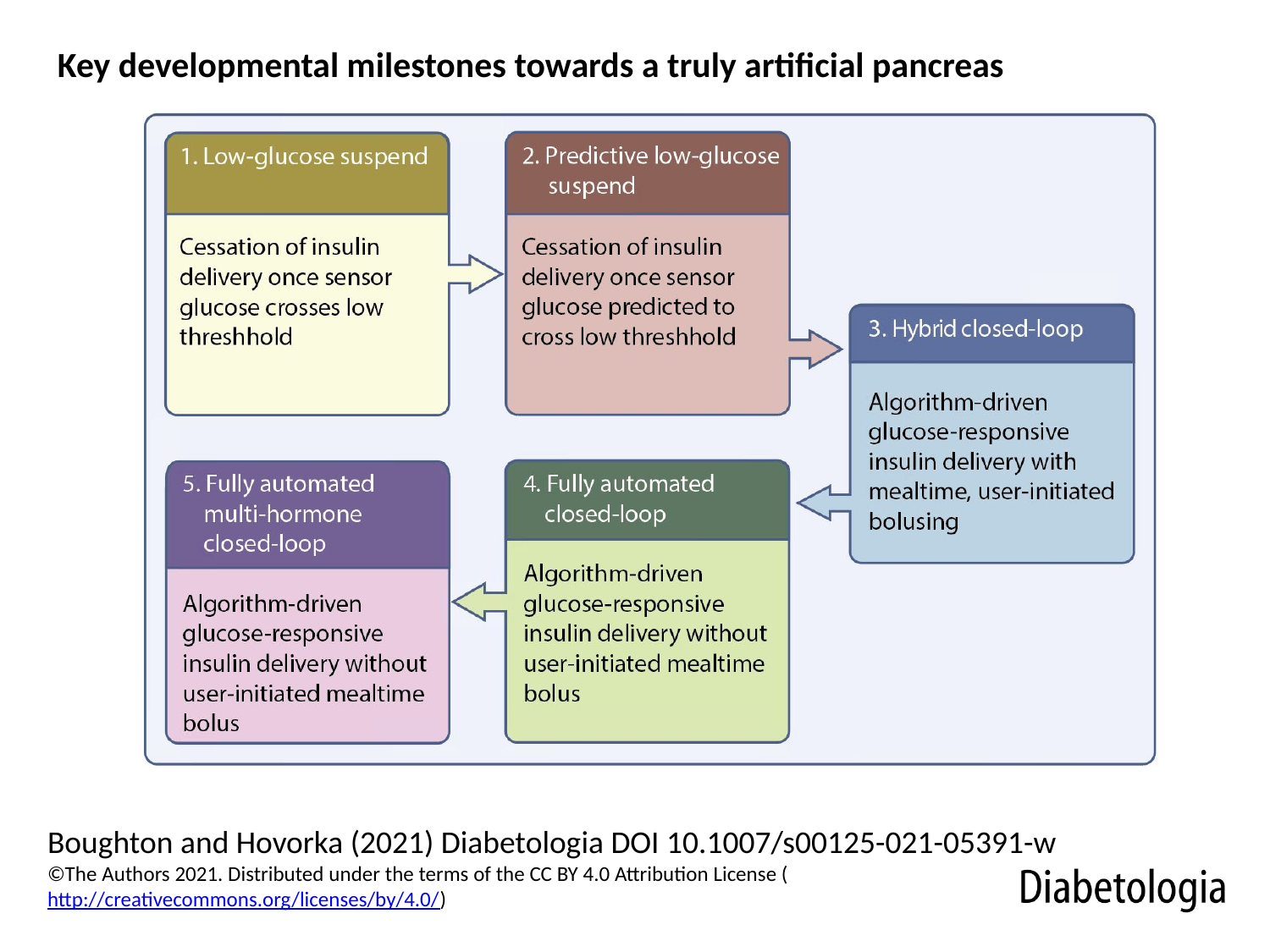

Key developmental milestones towards a truly artificial pancreas
Boughton and Hovorka (2021) Diabetologia DOI 10.1007/s00125-021-05391-w
©The Authors 2021. Distributed under the terms of the CC BY 4.0 Attribution License (http://creativecommons.org/licenses/by/4.0/)

## Slide 3
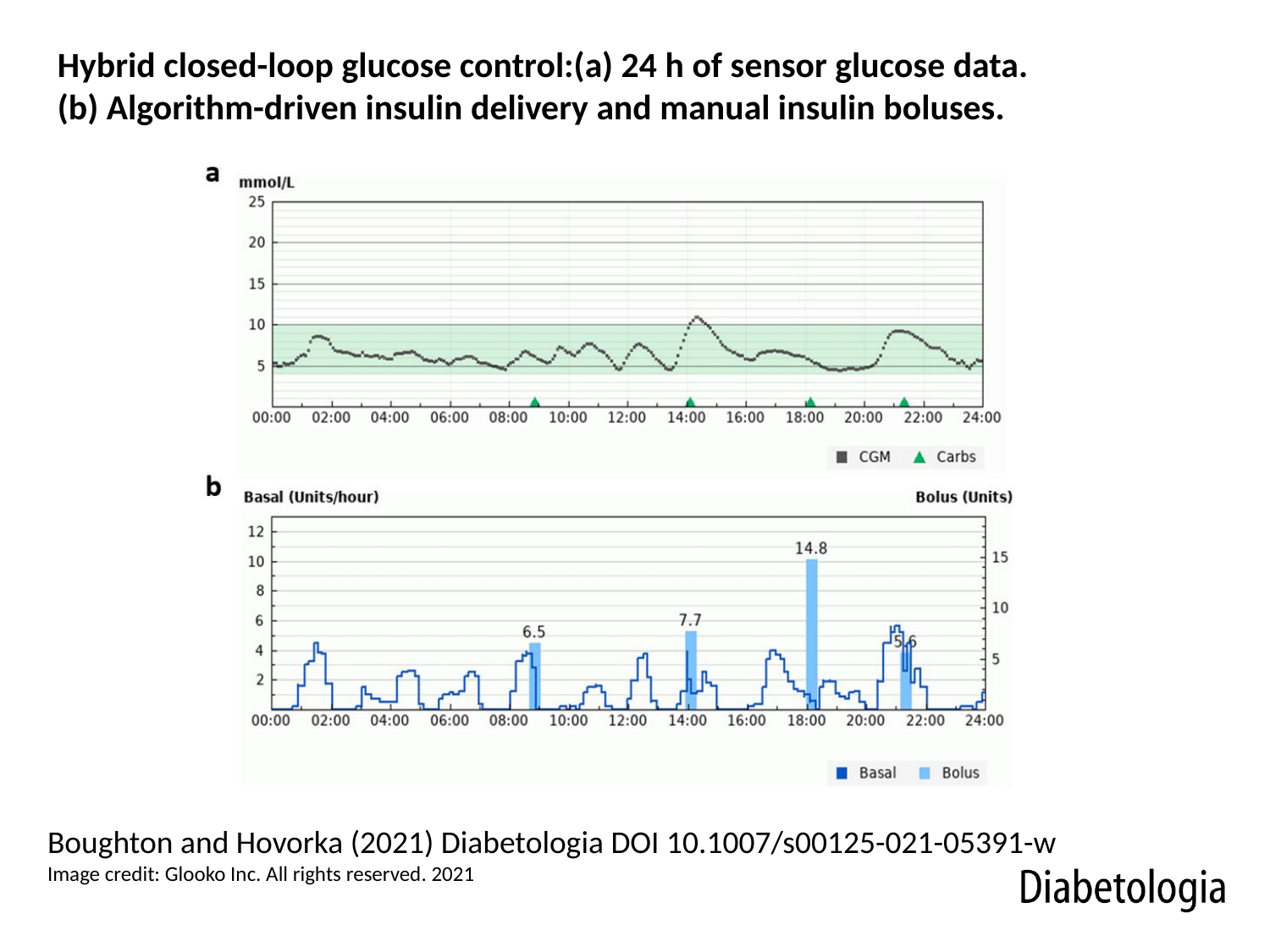

Hybrid closed-loop glucose control:(a) 24 h of sensor glucose data.
(b) Algorithm-driven insulin delivery and manual insulin boluses.
Boughton and Hovorka (2021) Diabetologia DOI 10.1007/s00125-021-05391-w
Image credit: Glooko Inc. All rights reserved. 2021

## Slide 4
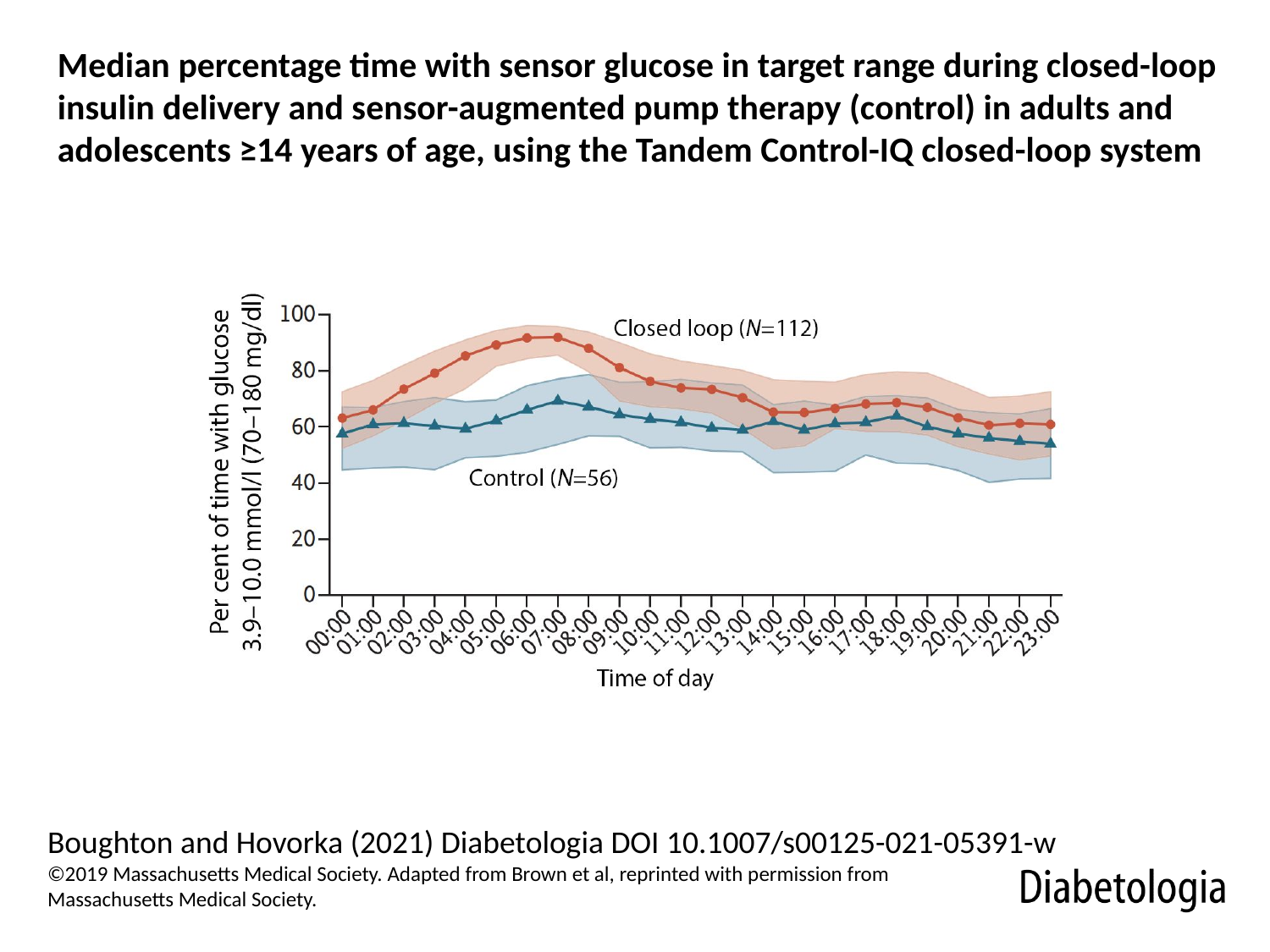

Median percentage time with sensor glucose in target range during closed-loop insulin delivery and sensor-augmented pump therapy (control) in adults and adolescents ≥14 years of age, using the Tandem Control-IQ closed-loop system
Boughton and Hovorka (2021) Diabetologia DOI 10.1007/s00125-021-05391-w
©2019 Massachusetts Medical Society. Adapted from Brown et al, reprinted with permission from
Massachusetts Medical Society.
